# Effect of Silicon Dioxide Nanoparticles on the Sintering Properties of Beta-Tricalcium Phosphate Composites

**DOI:** 10.3390/ma17040797

**Published:** 2024-02-07

**Authors:** Kazuaki Hashimoto, Masahiko Oiwa, Hirobumi Shibata

**Affiliations:** Department of Applied Chemistry, Faculty of Engineering, Chiba Institute of Technology, 2-17-1 Tsudanuma, Narashino-shi 275-0016, Chiba, Japan; masahikooiwa.sub@gmail.com (M.O.); hirobumi.shibata@p.chibakoudai.jp (H.S.)

**Keywords:** β-tricalcium phosphate, SiO_2_ particle dispersed β-TCP composite sintered body, densification, nanoparticle dispersion, polymerized complex method

## Abstract

Composite sintered bodies comprising silicon dioxide (SiO_2_) nanoparticles dispersed in β-tricalcium phosphate (β-TCP) were prepared. The addition of nano-sized colloidal SiO_2_ to the β-TCP produced well-dispersed secondary phase nanoparticles that promoted densification by suppressing grain growth and increasing linear shrinkage of the sintered bodies. The SiO_2_ was found not to react with the β-TCP at 1120 °C and the substitution of silicon for phosphorous to produce a solid solution did not occur. This lack of a reaction is ascribed to the absence of available calcium ions to compensate for the increase in charge associated with this substitution. The SiO_2_ nanoparticles were found to be present near the intersections of grain boundaries in the β-TCP. β-TCP composite sintered body containing 2.0 and 4.0 wt% SiO_2_ exhibited a bending strength comparable to that of cortical bone and hence could potentially be used as a bone filling material.

## 1. Introduction

In recent years, the demand for bone replacement materials used in the treatment of bone diseases has increased due to ongoing aging of the population. To date, β-tricalcium phosphate (β-Ca_3_(PO_4_)_2_: β-TCP) has been employed clinically for this purpose because this material shows excellent biocompatibility along with a high bioabsorbability and can replace autologous bone when implanted in a bone defect [1,2,3]. Even so, β-TCP has inferior osteogenic potential and lacks mechanical strength compared with autogenous bone. As an example, in the case that raw β-TCP powder is prepared via a solid phase reaction and subsequently sintered for 24 h at 1100 °C, which is below the alpha-beta phase transition temperature, grain growth can occur without an increase in density. The result is a sintered body that exhibits a flexural strength in the range of 40–60 MPa and a sintering ratio of approximately 80% [4]. It has been reported that sintered bodies having greater density and with bending strengths in the range of 150–200 MPa can be obtained by adding a trace oxide to prevent the crystalline phase transition when sintering at high temperatures of 1150 to 1250 °C [5,6]. However, the incorporation of these phase transition inhibitors may form a substitution-type solid solution with β-TCP, thus suppressing the original solubility of β-TCP and resulting in reduced bioabsorbability [7,8,9,10,11].

It has also been demonstrated that densification can be promoted by hot pressing [12,13], hot isostatic pressing [14,15] and spark plasma sintering [16,17], although the necessary equipment is expensive to purchase and to operate. The present work prepared composite sintered bodies in which nanoparticles were dispersed to generate a secondary phase [18,19]. Specifically, colloidal silica (SiO_2_) was added to β-TCP powder in the form of nanoparticles with the aim of improving the sintering characteristics and mechanical strength of the material.

Silicon is known to be essential for the growth and development of bone and cartilage [20] and is found at concentrations of the order of 100 ppm in bone and ligaments and 200–600 ppm in cartilage and other connective tissues [21]. The localized concentration of this element at active calcareous sites in the bones of young mice has also been noted, where it is involved in the early stages of biomineralization [22]. On the other hand, water-soluble Si has been shown to promote osteoblast proliferation, differentiation and collagen production under in vitro conditions and to act on osteoclasts in a dosage-dependent manner. Addition of 0–50 mM (0–1.4 ppm Si) orthosilicate to the medium of human osteoblasts was found to increase alkaline phosphatase and osteocalcin activity by 1.5- and 1.2-fold, respectively, suggesting enhanced bone formation, and type I collagen synthesis by 1.8-fold [23]. In addition, administration of 0.1–100 ppm water-soluble Si solution to the medium of human osteoblasts for 48 h increased osteoblast proliferation and cell differentiation in a dosage-dependent manner through upregulation of transforming growth factor beta (TGF-β) [24]. Furthermore, ionic products containing high concentrations of water-soluble Si of Bioglass^®^ are known to stimulate osteogenesis and collagen synthesis, and culturing with ionic products of Bioglass^®^ significantly increased proliferation, differentiation, collagen secretion, and survival of osteoblasts derived from rat cranial crown. Ionic products generated by Bioglass^®^ dissolution markedly increased genes encoding proteins involved in osteoblast proliferation, extracellular matrix remodeling, and extracellular matrix attachment, and had a marked effect on gene expression in human osteoblasts [25]. Similarly, ionic products of calcium silicate (CaSiO_3_) were observed to enhance blast cell activity [26]. In vitro experiments of rat osteoclast cultures treated with Si leached from silicon-containing calcium phosphate showed a dosage-dependent effect of Si on osteocytes and osteoclasts [27]. As reported above, many studies have reported that silicon activates osteoblast-like cells [28,29].

**Figure 1:**
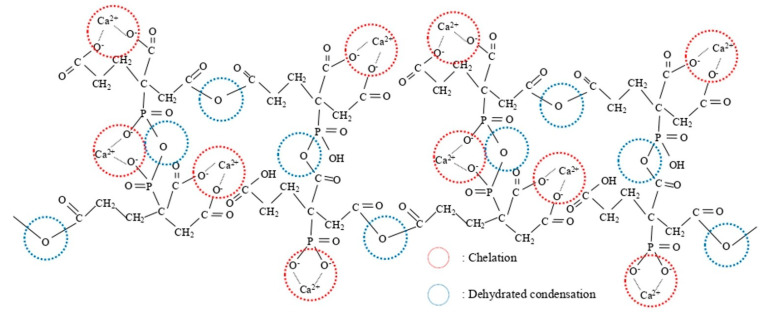
Predicted structure of polymeric gel obtained by polymerized complex method [30].

Another approach to producing high-density, high-strength sintered bodies is to utilize very finely powdered raw materials. Therefore, the present study also assessed the use of the polymerized complex method. In this process, metal ions are complexed, after which carboxyl and hydroxyl groups present in the complex are dehydrated and condensed by heating to prepare a polymeric gel (Figure 1) in which metal ions are coordinated [30].

This technique allows metal ions to be incorporated at an atomic level of dispersion and also permits the homogeneous dispersion of trace amounts of various additives. In addition, powders can be prepared rapidly and at low temperatures by sintering these gel precursors and hence the synthesis of raw material particles is facile [31,32].

In the work reported herein, composite sintered bodies incorporating well-dispersed silicon dioxide nanoparticles were prepared using fine β-TCP particles obtained by the polymerized complex method as the raw material. The effects of these nanoparticles on sintering characteristics and on the mechanical strength of the sintered β-TCP were investigated. 

## 2. Experimental

### 2.1. Preparation of β-TCP

Calcium nitrate tetrahydrate (Ca(NO_3_)_2_·4H_2_O, 99.0%, Fujifilm Wako Pure Chemicals Corporation, Osaka, Japan) was used as the Ca source and 2-phosphonobutane-1,2,4-tricarboxylic acid (PBTC, 50% in water, K-I Kasei, Tokyo, Japan) as the P source, respectively. A calcium nitrate solution was initially prepared by dissolving 21.98 g of Ca(NO_3_)_2_·4H_2_O in 50 mL of pure water with stirring. This solution was subsequently added to 33.02 g of the PBTC solution to prepare a mixture having a Ca/P molar ratio of 1.50. This mixed solution was subsequently heated with magnetic stirring at 150 °C for 3 h using a hot plate. The gel-like material obtained from the resulting dehydration-condensation polymerization was then transferred to a crucible and dried at 180 °C and 300 °C for 24 h and 10 h, respectively, to prepare the β-TCP precursor. This precursor was then calcined by heating to 900 °C at a heating rate of 5 °C·min^−1^ and held at that temperature for 6 h under atmospheric air.

The samples produced in this manner were assessed by X-ray diffraction (XRD) using a MiniFlex600 diffractometer (Rigaku, Tokyo, Japan) and by Fourier transform infrared (FT-IR) spectroscopy (FT-IR-4200, Jasco, Tokyo, Japan). Scanning electron microscopy (SEM; VE-7800, KEYENCE, Tokyo, Japan) was also used to observe the shapes and diameters of the particles comprising these materials while the particle size distributions were ascertained using a laser diffraction particle size analyzer (MT3300EXI3I, Microtrac Bell, Tokyo, Japan).

### 2.2. Preparation and Evaluation of Sintered Bodies

**Figure 2:**
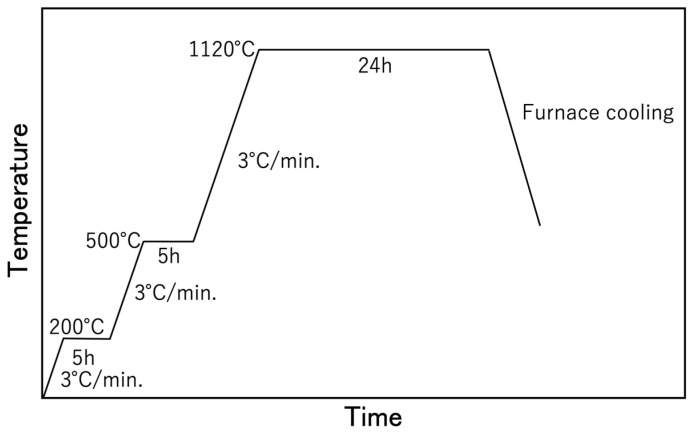
Heating rate for sintering in this work.

Fumed colloidal silica (SiO_2_; particle size: 12 nm, Strem Chemicals Inc., Newburyport, MD, USA) was added to the raw β-TCP powder at 0, 1, 2, 3, 4 or 5 wt% by external blending followed by mixing in ethanol for 12 h using alumina balls. The external blending refers to the mixing ratio (wt%) in which the weight of the raw material is taken as 100. After removing the ethanol with a rotary evaporator, the milled powder was mixed with a 2 wt% polyvinyl alcohol aqueous solution (Fujifilm Wako Pure Chemicals Corporation, Osaka, Japan), added at 8 wt%, and with deionized water, also added at 8 wt%, in a plastic bag. The resulting mixture was subsequently passed through a Teflon sieve having 200 μm pores. Kerosene (Fujifilm Wako Pure Chemicals Corporation, Osaka, Japan) was then added to the material at 5 wt% followed by thorough mixing in a plastic bag. The resulting mixture was placed in a cylindrical (11 mm dia.) mold forming machine (CDM-5M, Riken Kiki Corp., Tokyo, Japan) and subjected to a uniaxial pressure of 120 MPa for 1 min. This process fabricated a compact cylinder having a diameter of 11.0 mm and a thickness of 2.0 mm that was vacuum-sealed in a plastic bag using a vacuum packaging machine (V-280A, Tosei Corporation, Tokyo, Japan). The compact cylinder was then subjected to cold isostatic pressing (CIP) at 200 MPa for 10 min, employing an LCP-80–200A device (NPA System, Tokyo, Japan). The compact cylinders produced in this manner were sintered at 200 °C for 5 h, 500 °C for 5 h and 1120 °C for 24 h, applying a heating rate of 3 °C·min^−1^ between each temperature and under atmospheric air (Figure 2).

The sintered bodies were analyzed by XRD and FTIR spectroscopy. In addition, the lattice constants were obtained using the silicon internal standard method. Linear shrinkage was calculated as the difference between the length of the sample before sintering and the length of the sample after sintering divided by the length of the sample before sintering, expressed as a percentage. The porosity, bulk density and sintering ratios of the sintered bodies were ascertained using the Archimedes method. The microstructural observations of the sintered bodies were performed by field emission (FE) SEM (JSM-IT-800, JEOL Ltd., Tokyo, Japan), Energy Dispersive X-ray spectroscopy-attached SEM (EDX-SEM, JSM-7000, JEOL Ltd., Tokyo, Japan). The microstructural observations and elemental mapping were performed using scanning transmission electron microscopy (STEM, TalosF200X, FEI Company Japan Ltd., Tokyo, Japan). The specimens used for scanning electron microscopies were surface polished and then thermally etched at 1020 °C for 5 h. Furthermore, the test specimens used for scanning transmission electron microscopy observations were prepared as thin slice material using a focused ion beam machine (FIB NB5000, Hitachi High-Tech corporation, Tokyo, Japan). Finally, three-point bending tests (cross head speed,1 mm/min.) were carried out to assess mechanical strength with an Autograph AGS-X instrument (Shimadzu, Kyoto, Japan). The specimens used for the three-point bending test were cut to 3 mm × 4 mm × 40 mm and the cut surfaces were polished.

## 3. Results and Discussion

### 3.1. Evaluation of β-TCP as a Raw Material

**Figure 3:**
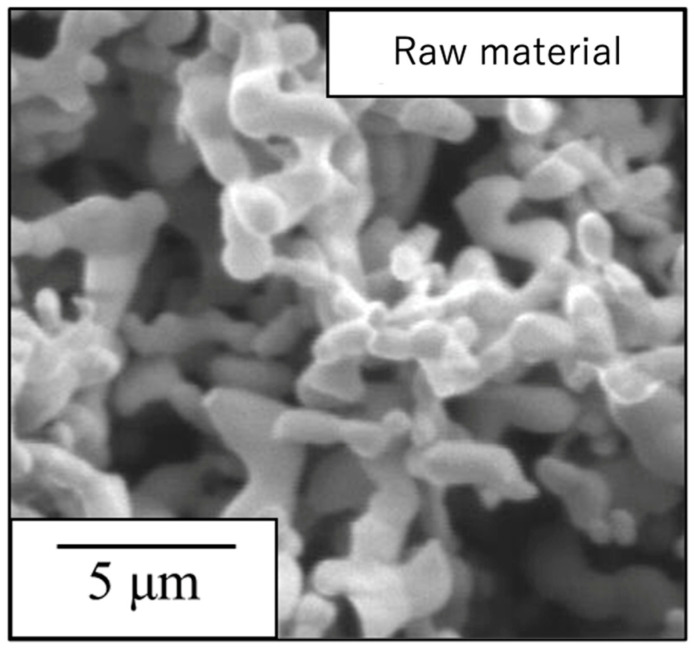
SEM image of the β-TCP used as the raw material in this work.

**Figure 4:**
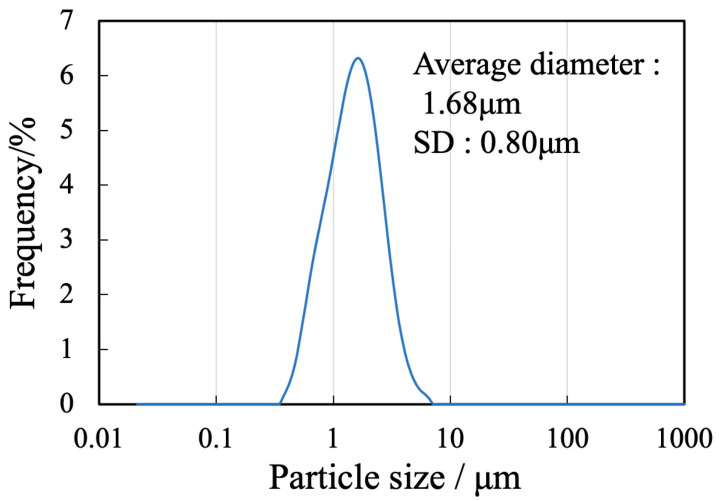
Particle size distribution of the β-TCP.

The sample generated by calcination of the precursor gel synthesized using the polymerized complex method with heating at 900 °C was found to comprise a single β-TCP phase based on analyses by XRD and FT-IR spectroscopy. The particle morphology and particle size distribution of this material were also examined. Figure 3 presents an SEM image of this β-TCP specimen after calcination while Figure 4 shows the particle size distribution data acquired after ball-milling of the precursor. The SEM image indicates narrow particles with sizes in the range of 2 to 5 µm. The size distribution of the particles obtained by ball milling ranged from 0.3 to 4 μm with a median diameter of 1.68 μm and SD of 0.80 μm. These results indicate that the raw material for the sintered bodies (that is, the β-TCP powder) consisted of fine particles having a relatively narrow range of sizes and an average size of less than 2 μm.

### 3.2. Preparation and Evaluation of β-TCP Composite Sintered Bodies Containing SiO_2_

**Figure 5:**
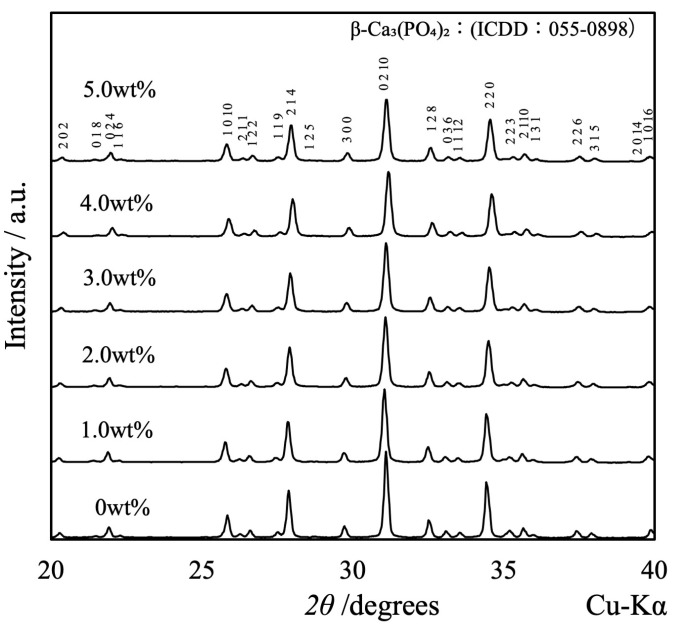
XRD patterns of β-TCP composite sintered bodies incorporating varying amounts of SiO_2_ nanoparticles.

Figure 5 shows the XRD patterns acquired from the β-TCP composite sintered bodies in which varying concentrations of SiO_2_ nanoparticles were dispersed. The peaks generated by each specimen were consistent with those of β-TCP (ICDD no. 055-0898), indicating that all samples had the same crystal structure as β-TCP [33]. Because the patterns of these sintered bodies were not modified, it appears that the SiO_2_ did not form a solid solution within the β-TCP crystal structure. Furthermore, no peaks related to SiO_2_ or various by-products were obtained, presumably because of the low concentration or amorphous nature of the SiO_2_. Interestingly, the XRD pattern generated by a specimen containing 10 wt% SiO_2_ contained peaks attributed to cristobalite (ICDD no. 001-043). This result suggests that the SiO_2_ nanoparticles added at lower concentrations were also changed to cristobalite upon heating.

**Figure 6:**
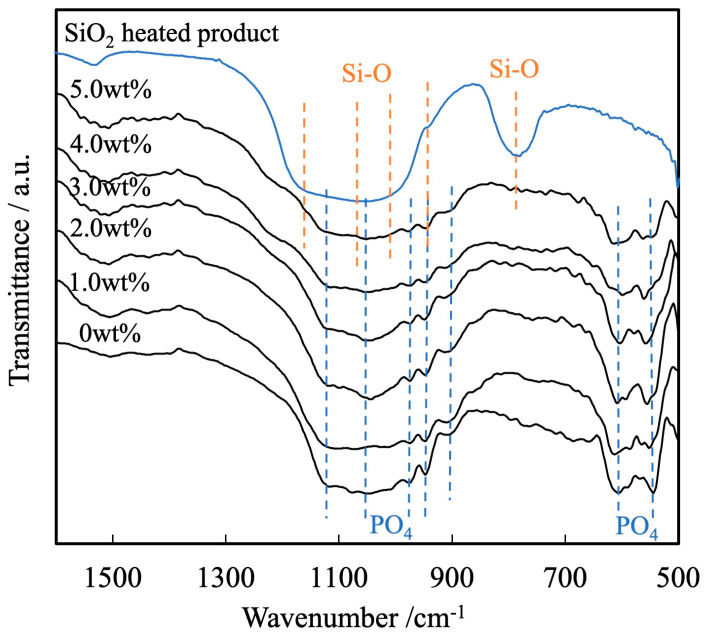
FTIR spectra of β-TCP composite sintered bodies incorporating varying amounts of SiO_2_ nanoparticles.

Figure 6 presents the FT-IR spectra of these same sintered bodies. The spectrum of a sample of heated colloidal SiO_2_ is also shown in the figure. Each of these spectra contains peaks ascribed to the bending and stretching vibrations of PO_4_ groups (attributed to the presence of calcium phosphate) within the ranges of 550 to 605 cm^−1^ and 905 to 1120 cm^−1^, respectively. In contrast, there are no peaks related to by-products such as hydroxyapatite or Ca_2_P_2_O_7_ [34,35]. In the FT-IR spectrum of the heated SiO_2_ sample shown in the figure, absorption spectra attributed to Si-O-Si stretching vibration and bending vibration of SiO_2_ were observed at 1000–1200 cm^−1^ and 800 cm^−1^, respectively [36,37], but as in the X-ray diffraction results, these absorptions could not be clearly distinguished. XRD patterns and FT-IR spectra showed no evidence of synthetic reactions such as compounding or substitution of the blended SiO_2_.

**Figure 7:**
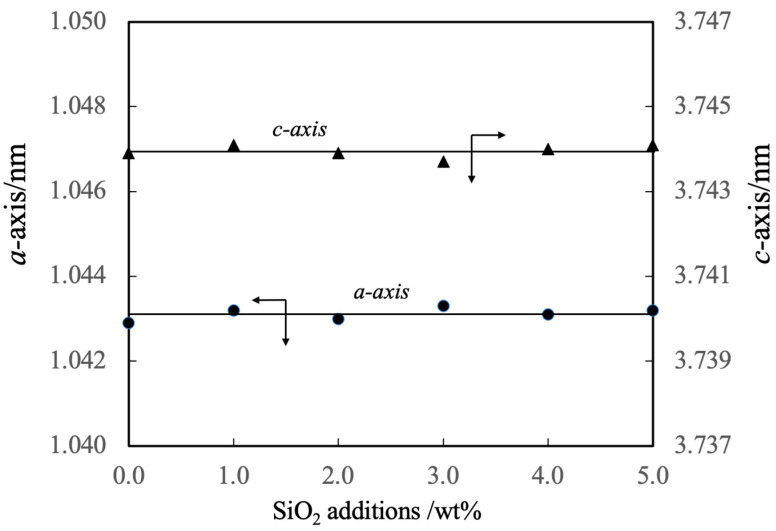
Lattice constants of β-TCP composite sintered bodies incorporating varying amounts of SiO_2_ nanoparticles. Closed circles and triangles indicate the lattice constants of the a-axis and c-axis, respectively. The direction of the arrow indicates the relationship between the axes in the figure.

The lattice constants of these specimens are summarized in Figure 7. These values indicate that the added SiO_2_ did not form a solid solution with the β-TCP. However, it should be noted that the authors previously prepared β-TCP in which silicon ions were substituted at phosphorus sites [38]. Although the composition of the β-TCP was not modified upon adding SiO_2_ in the present work, prior work demonstrated that the increased negative charge resulting from the replacement of PO_4_^3−^ with SiO_4_^4−^ ions was compensated for by the incorporation of Ca^2+^ ions. Hence, the Ca/P molar ratio was increased above the stoichiometric value of 1.50. In the present study, it is appears that silicon ions were not substituted into the β-TCP lattice because cations were not available to compensate for the associated change in charge.

The results so far suggest that the SiO_2_ added to β-TCP is present as SiO_2_ particles in the matrix of the sintered body.

### 3.3. Evaluations of Physical Properties

**Figure 8:**
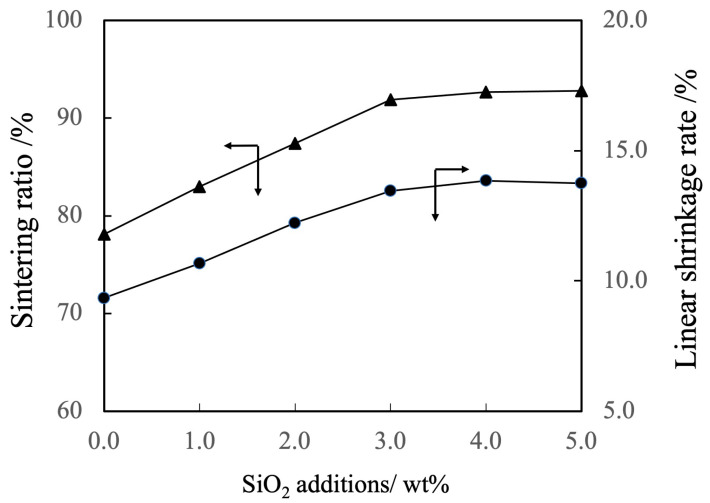
Sintering ratios and linear shrinkage rates of β-TCP composite sintered bodies incorporating varying amounts of SiO_2_ nanoparticles. Closed circles and triangles indicate linear shrinkage rate and sintering ratio, respectively. The direction of the arrow indicates the relationship between the axes in the figure.

Figure 8 shows the linear shrinkage and the sintered ratio (that is, relative density) of the various sintered bodies. The linear shrinkage of the pure β-TCP was 9.3% whereas this value was approximately 13% at a SiO_2_ addition level of 3.0 wt% and increased with increase of SiO_2_ addition. It was estimated that the increase in linear shrinkage due to sintering of SiO_2_ particle-dispersed β-TCP composite sintered bodies with increasing SiO_2_ addition was due to densification by discharging pores in the sintered body. The sintered bodies containing 3.0 wt% SiO_2_ showed the sintered ratio of 93% whereas the sintered ratio for the β-TCP was 78%. Hence, the sintered ratio also increased along with the SiO_2_ content.

**Figure 9:**
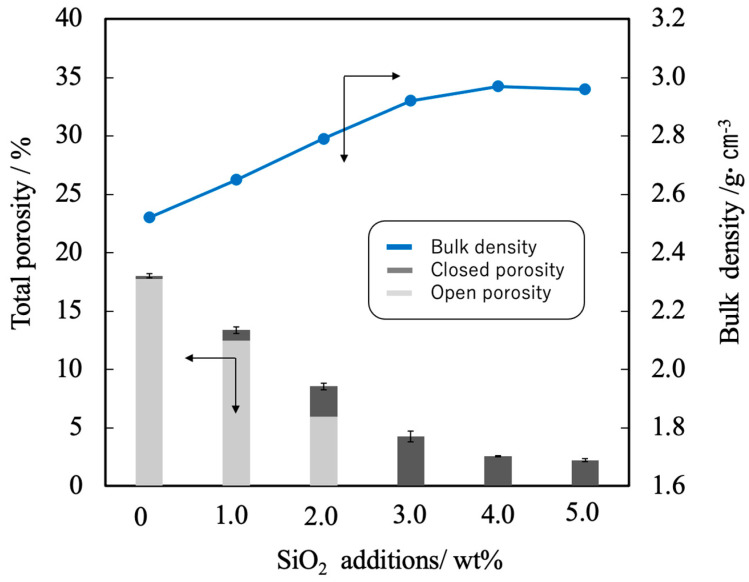
Bulk density and porosity values for β-TCP composite sintered bodies incorporating varying amounts of SiO_2_ nanoparticles. The closed circles and bar graphs show bulk density and porosity, respectively. The direction of the arrow indicates the relationship between the axes in the figure. Error bars indicate standard errors.

Figure 9 summarizes the porosity and bulk density values for sintered bodies. The bulk density of the β-TCP was 2.54, supporting the linear shrinkage and sintering data. In contrast, the bulk densities of the sintered bodies containing 3.0 wt% or more SiO_2_ were in the range of 2.95 to 2.98 g·cm^−3^ (compared with a theoretical density of 3.07 g·cm^−3^). These data demonstrate that the bulk density was increased with increases in SiO_2_ content in this range. Conversely, the open porosity values of the sintered bodies decreased with increasing SiO_2_ addition up to 2 wt% whereas the closed porosity values decreased with SiO_2_ addition above 3 wt%. The decrease of porosity indicates that the sintering characteristics of the material were improved. 

**Figure 10:**
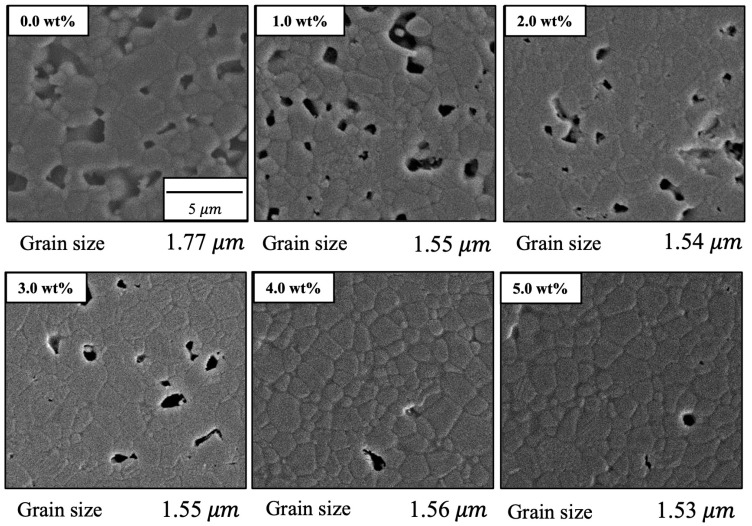
FE-SEM images of β-TCP composite sintered bodies incorporating varying amounts of SiO_2_ nanoparticles.

Figure 10 shows FE-SEM images of the various specimens. The particle sizes obtained from these images using the intercept method [39] are also provided in the figure. On this basis, it is apparent that both porosity and particle size were decreased with increases in the concentration of SiO_2_. These images are in agreement with the earlier porosity data because the open pores on the surfaces of the sintered bodies were similar to those measured by the Archimedes method, as described above. The grains in the pure sintered material were approximately 1.7 μm in size whereas SiO_2_ addition decreased this value to approximately 1.5 μm. These results confirm that porosity and grain size were decreased with increasing SiO_2_ content. Hence, the sintered bodies became denser with SiO_2_ addition. It is thought that SiO_2_ addition suppressed the diffusion of β-TCP, inhibited grain growth and promoted densification by discharging pores and restructuring the grain structure.

**Figure 11:**
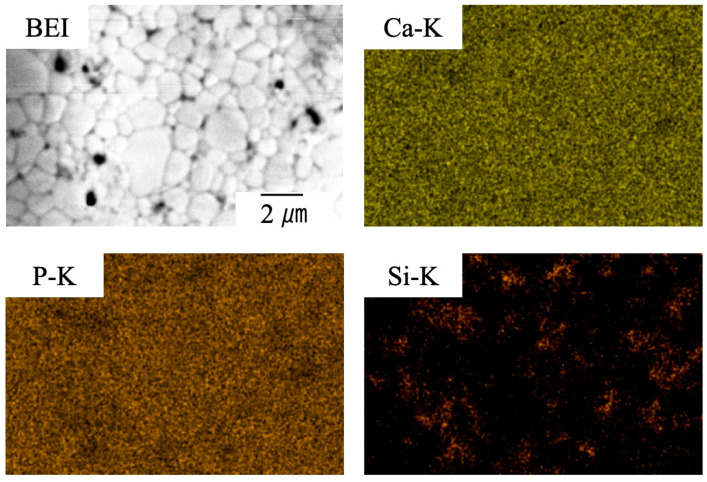
EDX-SEM images of β-TCP composite sintered bodies with 4.0 wt% SiO_2_ nanoparticles.

Figure 11 provides elemental mapping images of a sintered specimen containing 4.0 wt% SiO_2_ as acquired using EDX-SEM. These images show that Ca and P are present in the same locations and that Ca and P are absent where Si is present. In addition, Si is widely dispersed. These results provide more evidence that a solid solution was not formed between the β-TCP and SiO_2_ but rather that these materials existed as separate phases. However, it is not possible to determine from these images whether the SiO_2_ was located within the grains or at the grain boundaries.

**Figure 12:**
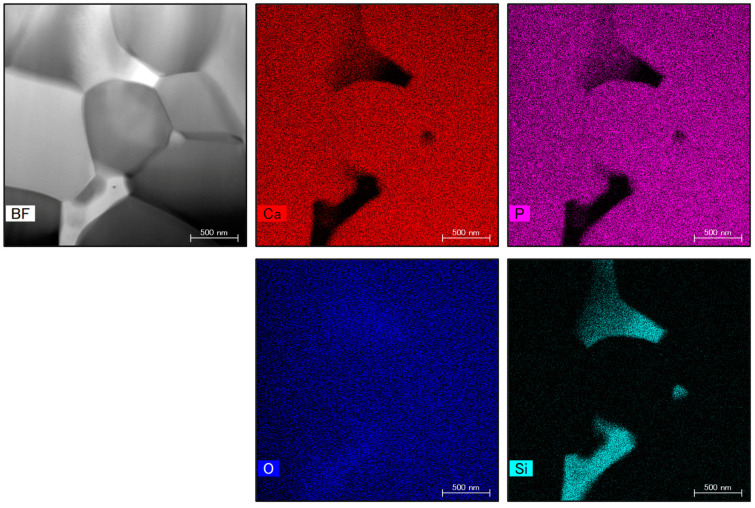
STEM images of β-TCP composite sintered bodies with 4.0 wt% SiO_2_ nanoparticles.

STEM was used for further high-resolution microstructural observations. Figure 12 presents STEM images showing the microstructure and elemental mapping of the 4.0 wt% SiO_2_ specimen. These image data revealed that the SiO_2_ nanoparticles ranged in size from 100 to 500 nm and were located at the intersection of grain boundaries.

### 3.4. Mechanical Strength Assessments

**Figure 13:**
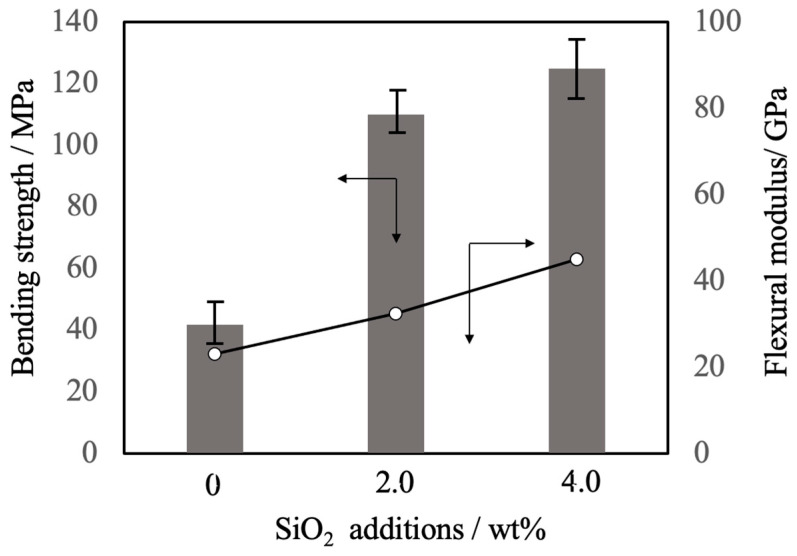
Bending strength and flexural modulus values of β-TCP composite sintered bodies incorporating varying amounts of SiO_2_ nanoparticles. The bar graph shows the bending strength, the opened circles the flexural modulus. The direction of the arrow indicates the relationship between the axes in the figure. Error bars indicate standard errors.

Figure 13 summarizes the three-point bending strength and flexural modulus values obtained for the sintered bodies. The average strength value for the pure β-TCP was 42 MPa while the specimens containing 2.0 and 4.0 wt% SiO_2_ had strengths of 110 and 125 MPa, respectively. Sintered bodies with 2.0 and 4.0 wt% SiO_2_ exhibited bending strengths comparable to that of cortical bone [40] and hence could possibly be used as a bone filling material. These increases in strength are attributed to densification and possibly to the suppression of crack propagation by SiO_2_ nanoparticles at the intersections between grain boundaries. The addition of these SiO_2_ nanoparticles resulted in higher values of mechanical strength that were more than comparable to those of β-TCP sintered bodies produced using the hot-pressing technique [12]. Because the hot-pressing specimens showed a higher degree of sintering than was obtained in the present work, the enhanced mechanical strength seen in this study likely resulted from both dispersion of the nanoparticles and densification [41].

**Figure 14:**
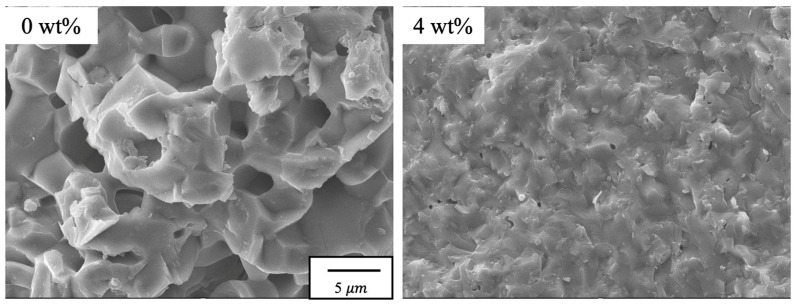
SEM images of the fracture surface of β-TCP composite sintered bodies with 4.0 wt% SiO_2_ nanoparticles and without.

SEM images of fracture surfaces of β-TCP composite sintered with and without 4.0 wt% SiO_2_ nanoparticles are shown in Figure 14. SEM images of fracture surfaces of sintered specimens without SiO_2_ nanoparticles showed a porous state and crack propagation at the grain boundaries around the pores, which in the case of this specimen was considered to be due to intergranular fracture. On the other hand, the sintered specimen with 4 wt% SiO_2_ nanoparticles was found to be pore-free and dense, indicating that crack propagation occurred via intragranular or intergranular fractures. In particular, the particles, which were considered to be SiO_2_ particles, were found to be widely distributed, suggesting that the crack propagation was suppressed by the particles.

Although there have been many evaluations of the mechanical strength of porous sintered β-TCP [42,43] and biphasic composites containing HAp [44,45], there are few reports on the subject of the mechanical strength of dense sintered β-TCP. Therefore, we consider the outcomes of studies such as the present study to be valuable data for bioceramics.

## 4. Conclusions

Composite sintered bodies comprising silicon dioxide nanoparticles dispersed in the microstructures of β-TCP were fabricated. Nano-sized colloidal silica was used as the silicon dioxide component in these materials. This silicon dioxide was well-dispersed as secondary phase particulates that suppressed grain growth in the sintered bodies and promoted densification by increasing linear shrinkage due to pore expulsion. However, substitution to form a solid solution with β-TCP was not observed even upon heating at 1120 °C. This lack of substitution likely can be attributed to the absence of free calcium ions required for charge compensation. Silicon dioxide was found to be present as 100–500 nm nanoparticles near the intersections of grain boundaries. Sintered bodies with 2.0 and 4.0 wt% SiO_2_ exhibited bending strengths comparable to that of cortical bone and hence could possibly be used as a bone filling material.

## Data Availability

Data are contained within the article.
